# The examination of the relationships among middle school students' epistemological beliefs, goal orientations, and achievement emotions: a structural equation modeling study

**DOI:** 10.1186/s40359-026-04488-4

**Published:** 2026-04-23

**Authors:** Barışcan Savaş, Mehmet Demirbağ

**Affiliations:** 1 Ministry of Education, Bursa, Turkey; 2 Department of Science Education, Faculty of Education, Bursa Uludag University, Bursa, 16059 Turkey

**Keywords:** Achievement emotions, Epistemological belief, Goal orientations, Middle school students

## Abstract

This study examined the relationships among middle school students' epistemological beliefs, achievement emotions, and goal orientations, and it was conducted with the participation of 1073 middle school students from seven different public schools in Turkey. A correlative research design was employed, and structural equation modeling (SEM) analysis was conducted. According to the results, sophisticated epistemological beliefs were positively related to mastery and performance goal orientations. In the relationship between epistemological beliefs and achievement emotions, it was observed that sophisticated epistemological beliefs in the dimensions of justification and source positively related to positive achievement emotions and negatively related to negative achievement emotions. On the other hand, it has been observed that sophisticated epistemological beliefs in the certainty dimension are negatively related to positive achievement emotions and positively related to negative achievement emotions. An analysis of the relationships between goal orientations and achievement emotions found no significant relationship between performance-approach goals and positive–negative achievement emotions, whereas there was a significant relationship between all other subdimensions. However, the study discussed inconsistent results for some subdimensions of epistemological beliefs and goal orientations. Further research is needed to examine the relationship between epistemological beliefs and achievement emotions in middle school students. This study is innovative in that it simultaneously tests the structural relationships between epistemological beliefs, goal orientations, and feelings of success in the context of science education using a large-scale sample. It addresses a gap in the literature by exploring how these constructs relate to each other within the same empirical framework.

## Introduction

Students' achievement in a subject is inevitably influenced by their emotional structures. Perceptions of achievement or failure and the basic emotions they feel in this situation, such as shame or pride, directly affect the goal related to the lesson in an attributional manner [58]. Recent studies emphasize that achievement emotions consist of multiple dimensions, including cognitive and motivational components [45, 59]. These components influence students' learning experiences in an integrated way and are central to understanding emotions in academic contexts [59].

One of these emotions, the emotion of achievement, is shaped by students' evaluations of their academic activities and the outcomes of these activities. According to Pekrun's [54] control-value theory, these emotions arise from the combination of the individual's subjective perception of control over activities and outcomes and the value that these activities and outcomes hold for the individual.

Many studies on achievement emotions have revealed that achievement emotions are intertwined with cognitive and motivational structures such as academic achievement in the domains of science and mathematics [52, 64], critical thinking and problem-solving [30, 61], teachers' preferred learning approaches [68], self-regulation, coping strategies [15], classroom engagement [22], and goal orientation [7]. A growing body of literature on these topics is available.

However, despite this relationship, the connection between achievement emotions and cognitive and motivational factors in general tends to be somewhat fragmented [55]. In recent years, theoretical claims have been made to combine theories and construct a "general theory of human emotions" to overcome the fragmented structure of achievement emotions and other motivational factors [56]. Conducting more studies that empirically test and demonstrate how achievement emotions intertwine with various structures to support these initiatives could contribute to theoretical discussions.

While some studies in the literature have tested the relationships between feelings of success and various variables using structural equation modeling (SEM) [25, 41], a comprehensive SEM study addressing feelings of success in the context of science education alongside epistemological beliefs and goal orientations has yet to be found. To address this theoretical gap, our study is based on the Control-Value Theory, which argues that feelings of success arise from an individual's subjective perceptions of control and value over their academic activities. Within this framework, the study assumes that epistemological beliefs represent an individual's perception of ‘control’ over the learning process (e.g., the perception of the mutability of knowledge), while achievement goals represent the ‘value’ placed on the subject being learned,thus, these two constructs are theorized as the fundamental antecedents of emotions. This theoretical pairing also provides an empirical basis for the General Theory of Human Emotions, which explains how cognitive and motivational structures integrate with emotions. Therefore, the main objective of this study is to test the structural relationships between middle school students' epistemological beliefs, goal orientations, and feelings of success in science class in light of the mechanisms predicted by the Control-Value Theory.

This study selected the achievement emotions and goal orientations of middle school students toward science courses. (1) Many cognitive and motivational factors emerge during the learning experience in this course, such as exam anxiety, emphasis on performance, beliefs about the nature and acquisition of knowledge, etc.; however, this also provides us with a natural field environment to understand and test many variables, such as epistemological beliefs, goal orientations, and emotions. 2) Achievement emotions have been studied empirically in the context of science education at the middle school level to a very limited extent [10].

### Literature review

#### Epistemological beliefs

A key concept in the field of education and cognitive psychology is epistemological beliefs. Epistemological beliefs are beliefs about the nature of knowledge and knowing [26]. When examining the historical development of epistemological beliefs in the field of educational psychology, three different approaches emerge.

The first approach is the developmental perspective, which began with Perry [62] and is based on the work of the researchers [32]. These researchers considered epistemological beliefs personal epistemology. They argued that personal epistemology progresses from a less developed to a more developed position, in accordance with the Piagetian perspective [63], through factors such as biological development and social interaction. According to developmental researchers, individuals' belief systems are classified from undeveloped to developed across all subfactors of beliefs about knowing and the nature of knowledge and are unidimensional [5, 26].

One of the pioneering researchers of the second approach, Schommer [67], took a different perspective from those who advocate a developmental view. According to Schommer [67], epistemological beliefs are more or less independent in nature and constitute a multidimensional structure divided into certainty of knowledge, simplicity of knowledge, source of knowledge, quick learning, and innate ability. Since the subdimensions of knowledge are independent characteristics, individuals can exhibit various degrees of each subdimension. According to Schommer [67], this classification falls within the contrast between naive and sophisticated. Individuals may hold different beliefs across various epistemological dimensions. For example, an individual may hold a naive belief in the certainty of knowledge (knowledge is certain) while having a sophisticated belief in the structure of knowledge (knowledge is a complex, interconnected structure).According to Hofer and Pintrich [26], four dimensions are clustered into two main directions: the nature of knowledge, comprising the certainty of knowledge and simplicity of knowledge, and the nature of knowing, comprising the source of knowledge and justification for knowing. Many empirical studies have used this four-dimensional structure to test the relationships between variables.

In the final approach, referred to as integrative perspectives, developmental stages from the developmental perspective (e.g., evaluativist) and dimensions from the multidimensional perspective (e.g., certainty of knowledge) are simultaneously utilized to understand epistemological beliefs within domain- and context-specific frameworks [42].

Researchers of this approach argue that epistemological beliefs are not independent of the subject and context but are specific to the domain, topic, and context [9, 24, 51]. For example, students can develop different beliefs in different areas, such as mathematics and social studies, based on their learning experiences [44]. In fact, students can develop specialized epistemological beliefs about any specific topic independently of their general beliefs [70].

Many researchers have shown that epistemological beliefs play an important role in learning and motivation outcomes such as academic achievement [71], self-efficacy [77], self-regulation [46], conceptual change [40], epistemic emotions [47], and achievement goals [11]. When closely examining the relationship between epistemological beliefs and goal orientation, it is expected that sophisticated epistemological beliefs would positively related to mastery-approach goals but negatively related to performance-approach goals (general expectation) [13, 43].

Specifically, in their examination of some studies, Kizilgünes et al. [33] reported that the advanced scientific epistemological beliefs of 6th-grade students positively related their mastery goal orientations. Using a sample of elementary and high school students, Chen [11] reported that students' advanced epistemological beliefs in the development and justification dimensions were positively related to their mastery goals but negatively related to their performance-avoidance goals. However, mixed results have been obtained in some studies. For example, in a study by Demirbağ and Bahçivan [14], the development and justification dimensions presented unexpected relationships with performance-approach goal orientations. Similarly, sophisticated epistemological beliefs in some dimensions are positively related to performance-approach goals [34, 39]. Even in studies where the general expectation was confirmed, such mixed results are also present [33, 74]. In summary, many studies have shown that epistemological beliefs are strong predictors of goal orientation components, and some results deviated from the expected patterns, such as positive correlations between advanced epistemological beliefs and performance-approach goals. However, to understand the existence of these results, further research may be needed on the achievement goals and epistemological beliefs of middle school students, particularly in the younger age group.

In this context, it can be argued that advanced epistemological beliefs may be positively related to both mastery-approach and performance-approach goal orientations. Furthermore, it can be expected that advanced epistemological beliefs may be positively related to mastery-avoidance goals and negatively related to performance-avoidance goals.

#### Achievement goals

Achievement goals are an important part of motivation theory, including definitions and evaluations of competence. The beliefs attributed to competence (how it is defined) and evaluations (how it is valenced) lead to the emergence of different achievement goals [18]. According to the theory, in the early years, competence was conceptualized as (1) understanding and mastering a task (setting standards and succeeding in them) and (2) performing better than others [3, 18, 48], with two dimensions: mastery goals and performance goals. Mastery goals involve individuals' approaches to competence related to mastery, whereas performance goals (performance approach) focus on normative comparisons, where individuals aim to perform better than others [18]. In subsequent years, psychologists proposed that competence was valenced in that it is either a positive, desirable possibility (i.e., success) or a negative, undesirable possibility (i.e., failure); this suggests that achievement goals can be recategorized into two categories: approach and avoidance [19]. Therefore, this 2 × 2 matrix presents the four-dimensional structure of achievement goals: mastery approach, mastery avoidance, performance approach and performance avoidance. According to Hulleman et al. [29], this fourfold structure focuses on achievement, failure, or avoidance in terms of interpersonal and intrapersonal characteristics. Avoidance and approach are intrapersonal (self-approach) characteristics of mastery goals, whereas performance goals stem from interpersonal (other approach) characteristics [17, 19]. Achievement goals are closely related to many cognitive and motivational concepts. For example, many studies have shown that achievement goals are closely related to self-regulation skills [16, 75], learning approaches [33, 73], cognitive engagement [66], epistemological beliefs [34, 37], and achievement emotions [7]. When the relationship between achievement goals and achievement emotions is closely examined, these two concepts are theoretically reciprocal. For example, it is assumed that the speed of progress toward achieving a goal leads to the experience of certain emotions. Conversely, emotional experiences affect achievement goals [35, 36]. According to Pekrun [54], emotions are particularly dependent on achievement goals that trigger and contribute to them. According to the theoretical assumption, mastery and performance approaches are generally associated with positive emotions, whereas avoidance orientations are associated with negative emotions [57]. In a comprehensive meta-analysis [7] of 312 empirical studies testing these theoretical assumptions, some unexpected results emerged,however, the theoretical connections between achievement emotions and achievement goals were largely confirmed. In other words, mastery-approach goals are related to activity emotions, performance-approach goals are associated with positive outcome emotions, and performance-avoidance goals are linked to negative outcome emotions [7].

When reviewing the literature examining these two variables, it can be expected that mastery and performance approach goal orientations will be positively related to positive achievement emotions and negatively related to negative achievement emotions. Additionally, it can be said that mastery and performance avoidance goal orientations will be positively related to negative achievement emotions such as anxiety and fear.

#### Achievement emotions

Achievement emotions are directly linked to achievement activities or achievement outcomes [54]. Activity emotions are experienced during participation in an activity (for example, solving a science problem). Outcome emotions include both prospective outcome emotions (for example, those related to potential achievements or failures) and retrospective outcome emotions (for example, those related to previous achievements and failures), and all three fall under the broader category of academic emotions [47]. Achievement emotions based on control–value theory are as follows: (1) subjective/internal control over achievement activities and their outcomes (e.g., the expectation that persistence in studying can lead to success) and (2) the subjective values of these activities and outcomes (e.g., the perceived importance of achievement) [54], p.317).

Additionally, achievement emotions arise from conceptualizing achievement activities or outcomes based on time (past, present, future) [59]. In this framework, pleasure (or enjoyment) is considered a positive activating emotion that arises when students positively evaluate their learning activities or outcomes. Anxiety is a negative activating emotion linked to the anticipation of failure or lack of control over learning tasks. Shame is a negative deactivating emotion associated with negative evaluations of one's abilities or efforts following failure [7, 54].

Recent studies on achievement emotions suggest that these emotions are closely related to others derived from control–value theory [55]. For example, achievement emotions, along with epistemic emotions, are included in the "Emotions" section of the "General Theory of Human Emotions." Given that epistemological beliefs are considered a core concept of epistemic emotions and many other cognitive and motivational concepts, they are also closely related to achievement emotions.

Therefore, the following hypotheses can be expected between these two variables.

For example, in domains where knowledge is more certain and unchanging, individuals' subjective control and values related to initiating and sustaining an activity and ultimately achieving success can influence the formation of different emotions. In more detail, physics knowledge is not certain, and knowledge is interconnected. A statement such as “I have not experienced this task related to physics, and I struggled. I might insist on this task or I might not, and as a result, I expect either achievement or failure” could trigger both positive and negative achievement emotions. In fields where knowledge is uncertain and interconnected (e.g., physics), the experience of struggling with a task and the dilemma of continuing or discontinuing the task can be expected to trigger both positive and negative feelings of success through the expectation of ultimate success or failure. In this context, having developed epistemological beliefs about the certainty and development of knowledge may be positively related to both positive and negative feelings of success and may yield mixed results.

In addition, an individual who sees themselves as the source of knowledge and holds the belief in evaluating evidence through multiple justifications (sophisticated epistemological beliefs) may experience positive emotions during and as a result of a science-related activity (such as problem-solving or engaging in argumentation) because the process aligns with and supports their epistemological belief. For example, positive emotions may be generated if individuals reach the best argument and succeed. Therefore, a positive relationship can be expected between (sophisticated) epistemological beliefs and positive feelings of success, while a negative relationship can be expected with negative feelings. Owing to such theoretical assumptions and hypothesis, we believe that epistemological beliefs may significantly impact achievement emotions, and we aim to test this possibility empirically.

Although epistemological beliefs and achievement emotions are grounded in different object foci, they intersect in meaningful ways within the learning process. Epistemological beliefs shape how students interpret learning tasks, evaluate their competence, and assign value to academic outcomes. These interpretations, in turn, influence the emergence of achievement emotions by altering students' sense of control and value, the two core components of the control-value theory. Therefore, even though the focal objects differ (knowledge vs. achievement), epistemological beliefs can significantly contribute to students' emotional experiences by acting as foundational cognitive-motivational antecedents.

#### Proposed model and hypothesis

**Fig. 1:**
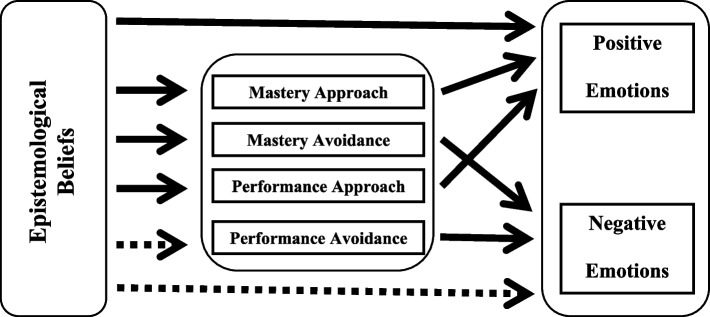
The proposed model of the study

Considering the aforementioned literature, the structural model in Fig. 1 was proposed. While the dotted lines represent a negative estimate, the solid lines represent a positive estimate in Fig. 1. The model suggests that middle school students' epistemological beliefs related to their goal orientations and achievement emotions. Additionally, goal orientations are related to achievement emotions.

First, since epistemological beliefs are a central concept [26], we placed them at the beginning of the model and drew a path from it to goal orientation and achievement emotions. As illustrated in Fig. 1, the proposed structural model begins with students’ epistemological beliefs, which are hypothesized to predict both their achievement goal orientations and achievement emotions. In this model, epistemological beliefs serve as antecedents, shaping how students value and perceive control over their learning. Achievement goal orientations, in turn, are proposed to mediate the relationship between epistemological beliefs and achievement emotions. Specifically, the model posits direct paths from epistemological beliefs to four types of goal orientations (mastery-approach, mastery-avoidance, performance-approach, performance-avoidance), and from each of these goal orientations to both positive and negative achievement emotions. Solid arrows in the model represent hypothesized positive relationships, while dotted arrows denote negative relationships.

Although the relationship between goal orientation and emotions is reciprocal, we identified a direction from goal orientation to achievement emotions, which is consistent with control–value theory [54], p.328) and many studies. First, mixed results can be expected when the relationship between epistemological beliefs and goal orientations is considered. Generally, a well-developed epistemological belief positively related to learning-oriented goal orientations, whereas avoidance goals are expected to be negatively related. However, owing to some of the studies mentioned above, as well as the high-stakes exams and competition in contexts such as Turkey, the results from our previous studies led us to revise our hypothesis.

H1-Sophisticated epistemological beliefs are expected to positively relationship mastery-and performance-approach goal orientations. For example, students with an advanced epistemology (e.g. Knowledge is tentative) may have goal orientations that align with their epistemological beliefs, such as a mastery approach to learning changing knowledge and demonstrate their performance to others (presenting multiple pieces of evidence). However, students with advanced epistemological beliefs may have mastery-avoidance beliefs, such as "Knowledge can change, I feel that I should control the learning process of these changing facts, I am afraid of not learning all of this knowledge." Therefore, sophisticated epistemological beliefs are expected to positively relationship mastery avoidance (H2). On the other hand, the relationship between epistemological beliefs and performance avoidance are expected to negative, which is consistent with previous studies (H3).

When examining the relationship between goal orientation and achievement emotions, it can be expected that mastery- and performance-approach goal orientations positively related to positive emotions and negatively related to negative emotions. For example, the goal of mastering an entire topic and being more successful than others can trigger positive emotions (H4). Similarly, since mastery- and performance-avoidance goal orientations are closely related to anxiety and fear, it can be expected that avoidance goal orientations related to negative emotions. For example, hesitating to understand the entire content of science lessons or fearing poor performance in front of others may positively related to negative emotions (H5).

Although no study has directly tested the structural relationships between epistemological beliefs and achievement emotions, some assumptions can be proposed. Similar to goal orientation, mixed results can be expected. In the theoretical framework section above, we present some speculations regarding our expectations.

However, having an advanced epistemological belief can generally enable participation in a science-related activity and positively evaluate the achievement status after the activity. In this case, it can be expected that a developed epistemological belief positively related to positive emotions and negatively related to negative emotions. However, based on the examples detailed in the literature section, having developed epistemological beliefs about the certainty and structure of knowledge may be positively related to both positive and negative feelings of success. Therefore, we propose the hypothesis that some dimensions of epistemological beliefs yield mixed results. (H6).

#### Research questions

Considering the aim of the study, the following research question was addressed:

1. What are the relationships among middle school students’ epistemological beliefs, achievement goals and achievement emotions?

## Method

In this study, a correlational research design [20] was used to investigate the relationships between epistemological beliefs, goal orientations, and achievement emotions. The study was conducted as a cross-sectional quantitative study. 

### Participants

The data for the study were collected from 1200 students studying in seven different public schools located in southeastern Türkiye. The schools were selected using a convenience sampling method, based on the researchers’ administrative access and permissions obtained from the local Directorate of National Education.

The inclusion criteria for participation were being enrolled as a 5th- to 8th-grade student and attending science classes as part of the national curriculum. After the data were collected, some students did not fill out the questionnaires adequately. Students who provided incomplete or inconsistent responses were excluded from the study. For this reason, 1073 students were included in the study.

### Instruments

The instruments consist of tree sections: the epistemological beliefs questionnaire, the goal orientations questionnaire, and the achievement emotions questionnaire.

These instruments were selected based on their strong theoretical grounding, widespread use in literature, suitability for middle school samples, and availability of validated language adaptations. Furthermore, these instruments were preferred because they are conceptually aligned with Control-Value Theory and structurally suitable for modeling multidimensional latent variables.

#### Scientific epistemological beliefs scale

The epistemological beliefs scale developed by Conley et al. [12] comprises four subdimensions: certainty of knowledge (e.g., scientific knowledge is always true), source (e.g., whatever the teacher says in the science class is true), justification (e.g., there can be more than one way for scientists to test their ideas), and development (e.g., ideas in science sometimes change). This scale was preferred because it operationalizes epistemological beliefs in a multidimensional and domain-specific manner, which is consistent with contemporary integrative perspectives in personal epistemology. Compared to earlier unidimensional or general belief scales (e.g., [67]), the Conley et al. [12] instrument provides a clearer distinction between the nature of knowledge and the nature of knowing, making it particularly suitable for science education research.

The scale comprises 26 items measured via a 5-point Likert scale (1 = Strongly Disagree to 5 = Strongly Agree), and the subdimensions of certainty, source, justification, and development include 6, 5, 9, and 6 items, respectively. Before the analysis, the items in the certainty and source dimensions were recoded so that higher scores on the scale in these dimensions corresponded to more advanced epistemological beliefs. Bahçivan [4] adapted the scale to Turkish using a sample of Turkish preservice science teachers and reported acceptable fit indices (χ2/df = 1.44, comparative fit index (CFI) = 0.95, Tucker‒Lewis index (TLI) = 0.93, and root mean square error of approximation (RMSEA) = 0.04) and acceptable alpha reliability scores ranging from 0.66 to 0.82.

The scale adapted by Özkan [49] for middle school students differs from that used in the study by Conley et al.,this is because elements from the domain of the nature of knowledge (certainty) merged with elements from the domain of the nature of knowing (source) [49]. This three-factor structure was examined via structural equation modeling (SEM) and confirmatory factor analysis (CFA). The results demonstrated the model's fit (GFI = 0.92, AGFI = 0.91, RMSEA = 0.06, S-RMR = 0.06). The reliability of the questionnaire is represented by a Cronbach's alpha coefficient of 0.76. The reliability of the "justification" dimension was found to be a Cronbach's alpha coefficient of 0.77, the "development" dimension had a Cronbach's alpha coefficient of 0.59, and the "resources/certainty" dimension had a Cronbach's alpha coefficient of 0.70. In this study, confirmatory factor analysis was conducted in accordance with the adaptation of Bahçivan [4] and by adhering to the four subdimensions of the scale; the results supported the four-factor structure. One item from the certainty dimension was removed because of a factor loading value lower than 0.3 [50]. The Cronbach's alpha coefficients for the epistemological beliefs questionnaire were 0.562 for the certainty subdimension, 0.588 for the source subdimension, 0.644 for the development subdimension, and 0.809 for the justification subdimension. The results of the confirmatory factor analysis (CFA) revealed fit indices (χ2/df = 2,54, GFI = 0.950, CFI = 0.914 and RMSEA = 0.038), demonstrating that the epistemological belief scale used in the present study has structural validity.

#### Achievement goal orientations scale

The Achievement Goal Questionnaire developed by Elliot and McGregor [18] is answered on a 5-point Likert scale ranging from 1 "Strongly Disagree" to 5 "Strongly Agree." It comprises 4 subscales and a total of 12 items. The mastery-approach goal comprises 3 items (e.g., "I want to learn as much as possible from this class"), the mastery-avoidance goal comprises 3 items (e.g., "I worry that I may not learn all that I possibly could in this class"), the performance-approach goal comprises 3 items (e.g., "It is important for me to do better than others"), and the performance-avoidance goal comprises 3 items (e.g., "I just want to avoid doing poorly in this class"). This scale was preferred because it includes avoidance goals, which are often missing in other tools but essential for examining negative emotions.

Şenler and Sungur [72] translated the scale into Turkish and conducted a validity study with 616 middle school students. The alpha coefficients for the sample are as follows: learning approach, 0.81, learning avoidance, 0.65; performance approach, 0.69; and performance avoidance, 0.64. The results of the confirmatory factor analysis supported the four-factor structure of the scale (GFI = 0.92, CFI = 0.92, NFI = 0.90, SRMR = 0.07) [72]. This study used the Goal Orientations Questionnaire to measure middle school students' goal orientations in science classes. The Cronbach's alpha coefficients for the Goal Orientations Questionnaire were 0.772 for the mastery-approach subdimension, 0.576 for the mastery-avoidance subdimension, 0.735 for the performance-approach subdimension, and 0.494 for the performance-avoidance subdimension. The results of the confirmatory factor analysis (CFA) conducted to validate the four-factor structure revealed fit indices (χ2/df = 3,30, GFI = 0.976, CFI = 0.960 and RMSEA = 0.046), which demonstrated that the goal orientation scale used in the present study has structural validity.

#### Achievement emotions scale

The Achievement Emotion Questionnaire (AEQ) was developed by Elliot and later adapted by Peixoto et al., [53] for students in grades 5–7 (ages 10–13). It is a five-point Likert scale adapted into Turkish by Alpaslan and Ulubey [2]. The AEQ was preferred because it has been validated for use with middle school students and offers clear subdimensions compatible with SEM analysis.

**Fig. 2:**
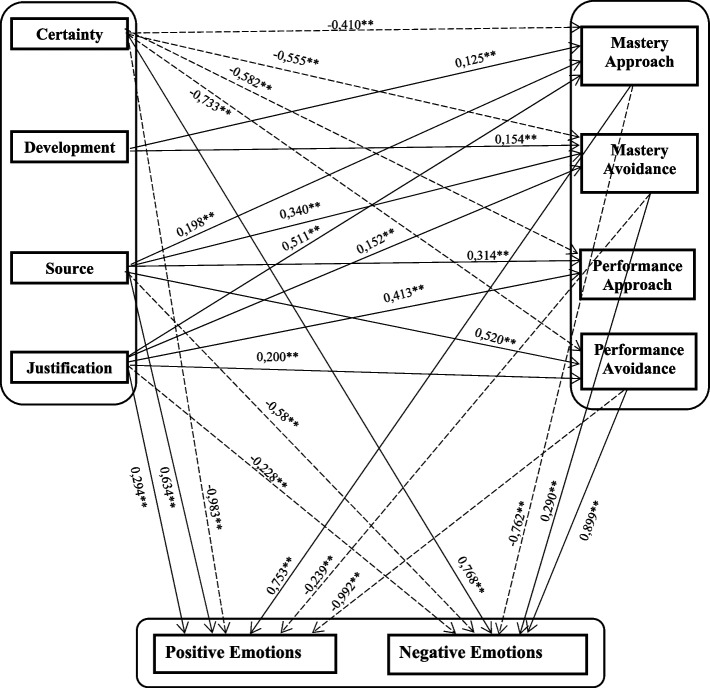
The Statistical Model (^*^p < 0.05, ^**^p < 0.001)

The scale has six dimensions: pride, enjoyment, anger, anxiety, hopelessness, and boredom. Each dimension comprises four items. CFA was conducted to ensure the validity of the data collected within the scope of the study. The CFA results were χ2 (df = 293, p = 0.000) = 697.28, SRMR = 0.050, RMSEA = 0.056, CFI = 0.93. The Cronbach's alpha coefficients for reliability are as follows: 0.80, 0.75, 0.84, 0.88, 0.88, and 0.77. In the present study, to measure middle school students' achievement emotions in science classes, the achievement emotions scale developed by Alpaslan and Ulubey [2] for mathematics classes was modified by replacing 'mathematics' with 'science' and then administered to middle school students. The six subdimensions of achievement emotions were reduced to two subdimensions for further analysis (such as SEM and others). In some studies, achievement emotions were reduced to positive (e.g., "I feel proud of my contribution in science class") and negative (e.g., "I feel tense in science class") emotions [69]. For this purpose, when conducting CFA, second-order factor analysis was used to reflect whether the six factors of the AEQ could be replicated and whether they fit the proposed model, as shown in Fig. 2. Four items from the negative emotions dimension was removed because of a factor loading value lower than 0.3 [50]. The emotions were grouped under the second-order factors of positive and negative emotions. The results of the confirmatory factor analysis (CFA) revealed fit indices (χ2/df = 4,71, GFI = 0.908, CFI = 0.932 and RMSEA = 0.059), demonstrating that the Achievement Emotions Scale used in the present study has structural validity. Finally, Cronbach's alpha values for these factors were 0.903 for positive emotions and 0.919 for negative emotions.

### Procedure

In this study, the necessary permission was obtained from a state university's Research and Publication Ethics Committee and, subsequently, from the Provincial Directorate of National Education of the city where the study was conducted. During the data collection phase of the study, students' voluntary participation was ensured. Before the study, the students' parents were informed about the study, and parental consent forms were obtained. The data collection process was conducted by the researcher, who visited schools and gathered data during a single class period. It took the students approximately one class period (40 min) to answer the questionnaires. Before the questionnaires were administered to the students, the researcher explained the aim and importance of the study and communicated that participation information would be kept completely confidential. The questions of the students who voluntarily participated in the study were answered immediately by the researcher, and necessary explanations were provided. The students were informed that they should read the questionnaire items carefully and independently from each other and that the voluntary participants could withdraw from the study at any time if they wished. The authors obtained permission from the scientists who developed the study's data collection scales. Additionally, the study results comply with the 1964 Helsinki Declaration and its subsequent amendments, as well as comparable ethical standards.

### Analysis

The relationships between epistemological beliefs, goal orientations, and achievement emotions were examined using Structural Equation Modeling (SEM). As explained in the instruments section, confirmatory factor analyses (CFAs) were conducted for each scale. After removing items with low factor loadings, the final structures of the scales were confirmed.

The data were entered into SPSS 23, and reliability analyses (Cronbach’s alpha coefficients) were conducted for each subscale to assess internal consistency. The results showed acceptable to moderate reliability levels, with some subdimensions (e.g., performance-avoidance) showing relatively lower coefficients, which were acknowledged as limitations in the interpretation of the model. Prior to testing the structural model, reliability analyses and confirmatory factor analyses (CFAs) were conducted for each scale, and model fit was evaluated using commonly recommended fit indices (χ^2^/df, CFI, TLI, RMSEA, GFI).

## Results

Structural Equation Modeling (SEM) analysis was conducted to examine the relationships between middle school students' scientific epistemological beliefs, goal orientations, and feelings of success. The analysis resulted in a statistically valid model with acceptable fit indices, except for CFI and TLI (χ^2^/df = 3.15, GFI = 0.857, CFI = 0.847, TLI = 0.838, RMSEA = 0.045 and SRMR = 0.085). The structural relationships obtained are presented below, linked to the hypotheses (H1–H6) in the Proposed Model.

When examining the structural relationships between epistemological beliefs and goal orientations, it can be said that the findings are generally consistent with the proposed hypotheses, except for the sub-dimension of certainty of knowledge. It was observed that sophisticated beliefs in the development, source, and justification sub-dimensions were positively related to mastery goals (approach and avoidance).

This supports the hypothesis that sophisticated epistemology positively related to mastery approach (H1) and mastery avoidance (H2) goal orientations. Furthermore, it was found that sophisticated epistemological beliefs in the dimensions of source and justification exhibit a positive relationship with performance goals (approach and avoidance). This result is consistent with the H1 hypothesis that sophisticated epistemological beliefs positively related to performance approach goal orientations.

It was also found that sophisticated beliefs in the certainty of knowledge sub-dimension (belief that knowledge can change) were negatively related to the four goal orientation sub-dimensions. This finding is consistent with the expectation that the certainty of knowledge sub-dimension would be negatively related to performance avoidance goals (H3), but it contradicts the other hypotheses.

When examining the relationship between epistemological beliefs and achievement emotions, it confirmed the expectation that, in addition to general expectations, mixed results (H6) could emerge in some sub-dimensions. It was found that sophisticated epistemological beliefs in the source and justification dimensions were positively related to positive emotions and negatively related to negative emotions. This supports the expectation that these beliefs strengthen positive emotions by enhancing the experience of success through self-regulated learning processes. However, it was determined that advanced beliefs in the certainty sub-dimension (belief that knowledge is changeable) are positively related to negative emotions and negatively related to positive emotions. This finding is consistent with the specific expectation (H6) that the constantly changing nature of knowledge may trigger negative emotions by reducing students' motivation or increasing their sense of uncertainty.

When examining the relationships between goal orientations and achievement emotions, it was found that mastery approach orientations are positively related to positive achievement emotions and negatively related to negative achievement emotions. This supports the part of H4 hypothesis regarding mastery approach goals. However, no significant relationship was found between performance approach goals and positive or negative achievement emotions.

When examining the relationship between mastery avoidance orientations and the emotional dimension, mastery avoidance orientations showed a negative relationship with positive emotions and a positive relationship with negative emotions. Performance avoidance goals, on the other hand, were found to be negatively related to positive emotions and positively related to negative emotions. These findings support H5 hypothesis that mastery and performance avoidance goals would be positively related to negative emotions such as anxiety and fear.

## Discussion

When interpreting the study's findings, the quality of structural model fit indices was evaluated collectively. Specifically, the RMSEA value of 0.045 (below 0.05), the χ^2^/df ratio of 3.15, and the GFI value of 0.857 indicate that the model falls within acceptable limits in terms of absolute and approximate fit. However, the CFI (0.847) and TLI (0.838) values remain below the more stringent cut-off criteria (≥ 0.95) proposed by Hu and Bentler [27], suggesting that the model does not exhibit excellent fit with respect to these indices.

The CFI and TLI values obtained from the confirmatory factor analyses conducted separately for each of the three variables were relatively high. Nevertheless, when all scales were tested simultaneously within a single SEM model, low reliability coefficients in certain subdimensions, particularly avoidance of performance (α = 0.494)—together with the increased complexity of the overall model, may have contributed to the observed decline in CFI and TLI values. Indeed, prior research indicates that relative fit indices such as CFI and TLI tend to decrease in complex, multivariate models [38, 76]. Empirical evidence further suggests that achieving the strict cut-off values proposed by Hu and Bentler [27] becomes increasingly difficult when factor loadings are relatively weak and model complexity increases. In this regard, Marsh et al. [38] emphasize that such cut-off values are often difficult to attain in appropriate practice and that relying on a single “gold standard” for evaluating model fit may be inappropriate.

In contrast, although the SRMR value of 0.085 marginally exceeds the strict cut-off proposed by Hu and Bentler [27], it falls within the acceptable range (0.08–0.10) recommended by Kline [31], suggesting that the model reproduces the observed relationships at a reasonable level. Accordingly, the model demonstrates acceptable absolute fit, while remaining open to improvement in terms of relative fit. For this reason, this issue is treated as a limitation of the study, and the relationships among the three variables are discussed using probabilistic and associative language,accordingly, the term “relationship” is used rather than prediction, and definitive predictive claims are avoided.

When examining findings on the relationship between epistemological beliefs and goal orientations, it was observed that sophisticated beliefs in the subdimensions of knowledge development, source, and justification were positively related to mastery goals (approach and avoidance). This finding is consistent with previous studies [33, 43, 74] showing that these beliefs are positively related to the internal conditions of self-regulated learning processes. Students' belief that knowledge can change, that sources can vary, and that it must be justified with evidence encourages them to invest in the learning process and adopt mastery-oriented goals. This situation is fully consistent with the theory proposed by Muis [43], which states that epistemological beliefs determine the internal ‘standards’ necessary for learning goals in self-regulated learning (SRL) processes. Students who view knowledge as changeable and evidence-based (sophisticated) adopt learning-approach (MAp) goals more readily because they set the ‘understanding’ standard higher, and this increases the depth of cognitive strategies [43]. This supports the hypothesis that there is a positive relationship with mastery approach (H1) and mastery avoidance (H2) goal orientations.

It has been found that sophisticated beliefs regarding the source and justification of knowledge also exhibit a positive relationship with performance goals (approach and avoidance). This result partially contradicts general theoretical expectations and findings in the literature (negative relationship). However, this is consistent with mixed findings from some recent studies, such as Demirbağ and Bahçivan [14], and Lin and Tsai [34], which report that advanced epistemological beliefs are positively related to performance goals, and it is also consistent with H1 hypothesis. This situation can be explained by the fact that in contexts such as Turkey, where high-stakes exams are a fundamental component of the education system, students develop a constructivist and evidence-based epistemology while also prioritizing the goal of proving their performance. While students' sophisticated epistemological beliefs are supported through activities such as argumentation and evidence-based discussion in science learning environments, they may also be motivated to pursue performance-approach goals in order to demonstrate their epistemic competence to their peers and teachers.

It was found that sophisticated beliefs regarding the certainty of knowledge (belief that knowledge is changeable) are negatively related to the four goal-oriented sub-dimensions. This finding is consistent only with the expectation of a negative relationship with performance avoidance goals (H3), but contradicts the other hypotheses. The unexpected negative relationships obtained in the dimension of certainty of knowledge (belief that knowledge is changeable) suggest that the constantly changing nature of knowledge is not always perceived positively by students. Some students may think, “If knowledge is constantly changing, there is no need to make an excessive effort to learn it,” and this situation may result in a decrease in both mastery and performance goals. Muis and Franco [46] emphasize that when knowledge is believed to be constantly changing, the impression that there is no fixed criterion for evaluating success may reduce students' performance expectations and effort. These findings show that epistemological belief sub-dimensions interact with goal orientations in different ways and that the tendency of beliefs to spread “unlimitedly” across contexts [1] can create new belief clusters.

When examining the structural relationships between epistemological beliefs and achievement emotions, it is consistent with the hypothesis (H6) that speculates on general and specific expectations**.** The fact that advanced beliefs regarding the source and justification of knowledge are positively related to positive emotions and negatively related to negative emotions suggests that epistemological beliefs shape emotions through self-regulated learning processes. Students with sophisticated epistemological beliefs may demonstrate higher self-regulation in initiating, sustaining, and completing science-related tasks, which may enhance their experiences of success and strengthen positive emotions such as pleasure and pride [23, 45].

Conversely, it is noteworthy that advanced beliefs in the certainty dimension of knowledge are positively related to negative emotions. The changing nature of knowledge, especially in abstract and complex science topics, can increase feelings of uncertainty and loss of control for some students. When the information presented does not match students' existing knowledge schemas or conflicts with their epistemic beliefs, it can trigger negative emotions such as disappointment, confusion, and anxiety [6, 47]. From the perspective of control-value theory, the belief in the changeability of knowledge may weaken the subjective sense of control over success in some students and thus increase negative emotions [54, 60]. This situation, which defined by Bendixen and Rule [6] as ‘epistemic doubt’, can cause instability and anxiety in individuals as a result of the conflict between existing beliefs and new information. The perception that knowledge does not have a fixed criterion of truth (sophisticated certainty) may have triggered feelings of subjective loss of control and uncertainty in some students, leading to expectations of failure and consequently negative feelings of anxiety and disappointment.

The relationship between goal orientations and achievement emotions is consistent with the fundamental assumptions of control-value theory and H4.. The fact that mastery-approach goals are positively related to positive emotions and negatively related to negative emotions indicates that students who focus on understanding the task and believe they can achieve learning goals experience more enjoyment, pride, and joy [35]. This finding supports the idea that mastery-approach goals may play a protective role emotionally.

Avoidance orientations (mastery and performance avoidance), on the other hand, show a positive relationship with negative emotions. This finding supports the H5 hypothesis that avoidance tendencies will be positively related to negative emotions such as anxiety and fear.

Students with mastery-avoidance goals may perceive the learning process as a threat because they are afraid of “not being able to learn the whole subject,” and this can increase emotions such as anxiety and worry. Similarly, performance avoidance goals, being closely related to the motivation to avoid appearing unsuccessful in front of others, set the stage for students to experience anxiety and embarrassment in exam and assessment situations [8, 21, 65]. These findings are consistent with previous meta-analytic evidence on the relationship between mastery-avoidance and negative emotions [7, 28] and once again highlight the emotional risks of avoidance-based goals.

The absence of a significant relationship between performance-approach goals and positive and negative achievement emotions may be consistent with the complex and context-sensitive findings in the literature. While some studies have found performance-approach goals to be associated with positive emotions, others have reported that this relationship is weak or neutral. The lack of a significant relationship in this study suggests that performance-approach goals may not produce a unidirectional effect in terms of emotions and that contextual factors (e.g., teaching practices, assessment format, classroom climate) may shape this relationship.

This finding parallels the view expressed by Huang [28] that Performance approach goals can be driven by both positive (success motivation) and negative (fear of failure) impulses,these opposing impulses can neutralize each other by bringing their effects on emotions closer together. Similarly, Linnenbrink and Pintrich [35] emphasize that the stress created by the effort to prove competence can overshadow the pride felt from success and that the relationship of this goal with emotions is highly context-sensitive.

## Limitations

This study has some methodological and contextual limitations. First, the research was conducted using a cross-sectional design. Therefore, although the paths reported in the structural model provide clues about the direction of relationships between variables, these paths cannot be interpreted as causal relationships; the results should be evaluated within the limitations of the structural relationships of the proposed model.

Second, the study variables were measured using self-report scales. Students' perceptions of their epistemological beliefs, goal orientations, and feelings of success may contain respondent bias (e.g., social desirability) and measurement error. Additionally, relatively low internal consistency (Cronbach's alpha) values were obtained in some subscales of the scales used. Specifically, the performance-avoidance subscale of the Goal Orientations Scale (α = 0.49) and the certainty (α = 0.56), source (α = 0.59), and development (α = 0.64) subscales of the Epistemological Beliefs Scale indicate that measurement error may be relatively high. Therefore, statistical results related to these sub-dimensions should be evaluated within the framework of measurement limitations revealed by relatively low internal consistency coefficients.

Thirdly, when examining the fit indices of the structural model, although the RMSEA and χ^2^/df values indicate that the model fits at an acceptable level (RMSEA = 0.045; χ^2^/df = 3.15), the CFI (0.847) and TLI (0.838) values fall below the stricter cutoff values (≥ 0.95) proposed by Hu and Bentler [27]. This indicates that the comparative fit of the model is not excellent and that the findings should be interpreted with certain limitations, particularly in terms of CFI and TLI.

Finally, the fact that the measurements were conducted in the context of science classes means that the results can primarily be generalized to the context of science education. Studies conducted in different subject areas (e.g., mathematics, social studies) will be an important next step in testing the cross-domain consistency of the relationships between epistemological beliefs, goal orientations, and achievement emotions.

## Implications

Since comparative fit indices such as CFI and TLI tend to decline in complex models with many variables and paths, submodels with fewer variables can be tested in future research. For example, fit indices can be tested with simpler models that examine only the relationship between epistemological beliefs and achievement emotions.

Future researchers may focus on the reciprocal relationship of structural variables. Although goal orientations are considered important precursors of achievement emotions in the literature, the reciprocal relationship where emotions can trigger achievement goals can also be examined.

On the other hand, more research is needed to demonstrate the structural relationship between epistemological beliefs and achievement emotions. While the relationship between epistemological beliefs and epistemic emotions has generally been established, the relationship with achievement emotions, which differ in terms of object focus, remains to be clarified.

Some dimensions of epistemological beliefs have produced mixed results in recent years, and certain achievement goals have led to unexpected outcomes owing to their theoretical structure, which requires closer examination. Specifically, the certainty dimension of epistemological beliefs has yielded surprising results related to both goal orientations and achievement emotions. The relationships involving the mastery-avoidance dimension show similar patterns. In this context, there may be a need for studies that conduct in-depth qualitative research and measure emotions via different protocols.

Data from countries such as Türkiye, where Eastern and Western cultures blend, and from Asia–Pacific countries, are important. However, different samples and results from various countries are needed. According to Pekrun [55], for the theoretical integration of motivation in science, more research is needed to adequately consider the specificity of populations, situational conditions, and sociocultural contexts, including their differentiation.

## Conclusions

The findings indicate that the four sub-dimensions of epistemological beliefs are meaningfully related to mastery goals. Sophisticated epistemological beliefs regarding the source, development, and justification of knowledge are positively related to both mastery approach and mastery avoidance goals; while sophisticated beliefs regarding the certainty of knowledge negatively related to both mastery approach and mastery avoidance goals.

When examining the relationships between epistemological beliefs and performance goals, source, justification, and certainty dimensions, were found to related with performance goals in a meaningful way. Sophisticated beliefs in the source and justification dimensions are positively related to both performance-approach and performance-avoidance goals, while sophisticated beliefs in the certainty of knowledge dimension negatively related to the same goals. This pattern indicates that epistemological belief dimensions exhibit relationships with mastery and performance-based goals in different directions and strengths.

The structural relationships between epistemological beliefs and achievement emotions are also significantly related. Sophisticated beliefs in the dimensions of knowledge source and justification related to positive emotions positively and negative emotions negatively. In contrast, sophisticated beliefs in the dimension of certainty of knowledge show a negative relationship with positive emotions and a positive relationship with negative emotions. This finding suggests that beliefs about the changeability of knowledge may be accompanied by more intense negative emotions in some students, along with feelings of uncertainty and loss of control.

In terms of the relationships between goal orientations and achievement emotions, only the mastery approach related to positive emotions positively. Mastery avoidance and performance avoidance goals related to positive emotions negatively and negative emotions positively. Furthermore, the fact that the mastery approach is also positively related to negative emotions indicates that mastery-based goals may not always be associated with a protective pattern in emotional terms and shows that mastery goals can be experienced in some cases alongside pressure to succeed or anxiety about not being good enough.

## Data Availability

The data are publicly available at [https://doi.org/10.7910/DVN/HYZ6QH].
